# An increase in a long noncoding RNA ANRIL in peripheral plasma is an indicator of stable angina

**DOI:** 10.1016/j.clinsp.2023.100289

**Published:** 2023-10-16

**Authors:** Yunjuan Jiao, Fanming Meng, Gaoen Ma, Hetian Lei, Junwen Liu

**Affiliations:** a Department of Histology and Embryology, School of Basic Medical Sciences, Central South University, China; b Department of Pathology, School of Basic Medical Sciences, Xinxiang Medical University, China; c School of Forensic, School of Basic Medical Sciences, Central South University, China; d Department of Ophthalmology, The First Affiliated Hospital of Hainan Medical University, China; e Shenzhen Eye Hospital, Jinan University, Shenzhen Eye Institute, China; f China-Africa Research Center of Infectious Diseases, School of Basic Medical Sciences, Central South University, Hunan Province, China

**Keywords:** ANRIL, Coronary heart disease, Acute coronary syndrome, Stable angina, Myocardial infarction

## Abstract

**Introduction:**

Stable angina develops during physical activity or stress, and it is typically an aspect of Coronary Heart Disease (CHD) that can lead to arrhythmia, heart failure and even sudden death. ANRIL, an Antisense Noncoding RNA gene in the INK4 Locus, is associated with multiple disorders including CHD; however, expressional levels of ANRIL in between patients with stable angina and myocardial infarction, one of the acute coronary syndrome, have not been clarified yet.

**Methods:**

The authors enrolled 62 patients with myocardial infarction and 59 with stable angina before primary percutaneous coronary intervention, as well as 48 healthy volunteers. Their peripheral blood was collected for analysis of ANRIL and cardiac troponin I, a traditional diagnostic index of CHD by real-time PCR.

**Results:**

The data showed that ANRIL is a better diagnostic indicator than cardiac troponin I in patients with stable angina and that the levels of ANRIL are higher in patients with stable angina than those with the myocardial infarction.

**Discussion:**

The levels of ANRIL in peripheral plasma could be used as a good biomarker for stable angina.

## Highlights

• ANRIL is a better diagnostic indicator than cardiac troponin I in patients with stable angina.
• The levels of ANRIL are higher in patients with stable angina than those with myocardial infraction.
• The levels of ANRIL in peripheral plasma could be used as a good biomarker for stable angina.

## Introduction

A major characteristic of atherosclerosis is the formation of artery plaque that can narrow arteries and lead to Coronary Heart Disease (CHD). Stable angina is the clinical feature of the early stage of CHD, whereas myocardial infarction, one of acute coronary syndrome, is a late stage of CHD.^1^ The heart not getting enough oxygen is the direct cause of stable angina, and people with stable angina have symptoms usually when they are emotionally stressed or doing physical activity. In myocardial infarction, there was thrombosis formation or plaque rupture on the basis of the plaque. In the clinic, due to most of the patients diagnosed at the stage of acute coronary syndrome, the mortality risk is increased, so it is essential to identify biomarkers in the early stage of the CHD for early intervention to reduce mortality.

The long noncoding RNA (lncRNA), an important class of regulatory molecule involved in a myriad of biological processes,^2^^,^^3^ is often dysregulated in various pathological conditions and has been reported as biomarkers for the diagnosis of multiple diseases.^4^, ^5^, ^6^, ^7^ The lncRNA antisense noncoding RNA in the INK4 Locus (ANRIL) is encoded by the chromosome 9p21 region, and ANRIL is associated with coronary artery disease identified through Genome-Wide Association Studies (GWAS). The effects of ANRIL on the progress of atherosclerosis include regulation of vascular function, activation of macrophages, lipid metabolism, and inflammation, and the immune response.^8^^,^^9^ Zhou et al. showed that ANRIL is highly expressed in atherosclerotic plaques, and it may regulate the NF-κB pathway by acting as an antisense regulator to the CDKN2B-CDKN2A gene.^10^, ^11^, ^12^, ^13^

ANRIL is associated with CHD, but whether there is a difference in its expression in patients between stable angina and myocardial infarction remains elusive. The present investigation showed that ANRIL in the plasma of patients with stable angina increased more remarkably than the traditional index: cardiac troponin I, suggesting ANRIL can be used as a diagnostic biomarker for stable angina.

## Material and methods

### Patients, treatment and ethics

62 patients with myocardial infarction, 59 patients with stable angina, and 48 healthy volunteers (without apparent signs of cardiovascular disease) were enrolled in the third affiliated hospital of Central South University from September 2015 to May 2016. 5 mL blood was collected in anticoagulant tubes before any medical treatment; the blood was then centrifuged at 3000 rpm/min for 20 min, and the supernatant as plasma was carefully transferred into an RNase-free tube for RNA extraction.^5^^,^^6^

This study followed the ARRIVE guidelines and was approved by the Central South University ethics review board, all the patients and volunteers were informed and consented, and the study was performed to conform to the declaration of Helsinki. The approval number for the scientific research project by the Medical Ethics Committee of Xiangya Third Hospital, Central South University is 2014-S093.

### RNA isolation and real-time PCR

Total RNA was isolated using RNA serum/plasma kit according to the manufacturer's instructions. In brief, 1 mL of QIAzol Lysis Reagent to 400 μL of serum was added, mixed well, and then incubated at room temperature for 5 min (min). Subsequently, 400 μL of chloroform was added and vortexed vigorously for 15 s (s). After incubation at room temperature for an additional 3 min, the samples were centrifuged at 12,000g for 15 min at 4°C. The upper aqueous phase was transferred to a new EP tube with 1.5 times the volume of ethanol (anhydrous) and mixed well. 700 μL of the mixture was transferred to an RNeasy MinElute spin column for centrifugation at 8000g for 15s. The liquid part was then discarded, and the centrifugation step with the remaining mixture was repeated. 700 μL of buffer RWT to the column was added for centrifugation at 8000g for 15s, and the supernatant was discarded.

Next, 500 μL of Buffer RPE to the column was added for centrifugation at 8,000g for 15s, the pellet was kept for 500 μL of 80% ethanol, centrifugation at 8000g for 2 min. The RNeasy MinElute spin column was centrifuged again at 8000g for 5 min to ensure thorough drying of the membrane and then dried at the air at room temperature for 3 min.

The RNeasy MinElute spin column was then placed in a new 1.5 mL EP tube, and 14 μL of RNase-free H2O was added to the center of the membrane, and incubated at room temperature for 10 min. Finally, the column was centrifuged at 12,000g for 1 min to obtain the RNA.

To analyze ANRIL, RNA was reverse transcribed with GoScript^TM^ Reverse Transcription System, and real-time PCR was performed using GoTaq® qPCR Master mix. The primers used in this study were as the followings: ANRIL: 5′-CTGGGACTACAGATGCACCAC-3′(forward), 5′-GGAGGGAGCATG TCTGTTTCT-3′(reverse); GAPDH (as an internal control): 5′-GGGAGCCAAAAGGGTCAT-3′(forward), 5′-GAGTCCTTCCACGATACC AA-3′(reverse). The expression level of ANRIL was normalized to GAPDH using the 2^−ΔΔCT^ method. Each specimen underwent PCR testing three times^5^^,^^6^

### Biochemical analyses

Low Density Lipoprotein (LDL), High Density Lipoprotein (HDL), Triglyceride (TG), Creatine Kinase-MB (CK-MB), cardiac troponin I, and blood glucose were measured using a Hitachi 7600-010 fully automatic biochemical analyzer.^5^^,^^6^

### Statistical analyses

Data is described as means ± Standard Deviation (SD) and median for general characteristics of subjects. Spearman rank correlation was used to evaluate the association between levels of lncRNAs and continuous variables, all statistical analyses were performed using the SPSS 18.0 software. Differences between different groups were assessed using the One-Way ANOVA comparison method. A value of *p <* 0.05 considered to indicate statistical significance. The Receiver Operating Characteristic (ROC) curve was established to evaluate the predictive power of circulating ANRIL between the health and CHD subjects. To evaluate the prognostic value of lncRNAs, Receiver Operating Characteristic (ROC) curves were constructed, and the Area Under the ROC Curve (AUC) was determined. Reclassification analyses were performed to determine the additive value of lncRNAs to clinical parameters and other biomarkers.^5^^,^^6^

### Ethics

This study was approved by Central South University ethics review board, all the patients and volunteers were informed and consented, and the study was performed to conform to the declaration of Helsinki.

## Results

### Characteristics of the study population

Table 1 provides the demographic and clinical characteristics of the study population. A total of 121 patients participated in this study, with 59 patients diagnosed with stable angina and 62 patients diagnosed with acute myocardial infarction, based on the 2019 ESC Guidelines for the Diagnosis and Management of Chronic Coronary Syndromes and the 2017 Guidelines for the Diagnosis and Treatment of Acute ST-Segment Elevation Myocardial Infarction. The median ages for the stable angina and acute myocardial infarction groups were 65.29 ± 9.60 years and 68.25 ± 12.18 years, respectively. The control group included 48 healthy volunteers with a median age of 63.59 ± 6.28 years. This study examined the risk factors associated with coronary heart disease and the markers closely related to myocardial infarction in the blood of the three patient groups. The results showed no statistically significant differences in the levels of LDL, HDL, TG, and CK-MB between stable angina patients and acute myocardial infarction patients. However, the cTnI level in the blood of stable angina patients was 0.093 ± 0.28 ng/mL, significantly lower than that of acute myocardial infarction patients, which was 3.273 ± 2.85 ng/mL (*p <* 0.05).

**Table 1:** Study population characteristics.

Characteristics	Health (*n =* 48)	Angina (*n =* 59)	MI (*n =* 62)	p (MI/Angia)
Age (years)	63.59 ± 6.28	65.29 ± 9.60	68.25 ± 12.18	0.316
Male/female (n/n)	26/22	31/28	36/26	0.334
LDL (mmoL/L)	1.79 ± 0.93	2.01 ± 0.52	2.87 ± 0.97	0.402
HDL (mmoL/L)	1.04 ± 0.35	1.21 ± 0.42	1.90 ± 0.23	0.450
TG	1.39 ± 1.65	1.30 ± 0.53	1.00 ± 0.35	0.131
CK-MB	22.33 ± 6.66	85.8 ± 8.98	484.1 ± 128.1	0.360
cTnI(ng/mL)	0.060 ± 0.00	0.093 ± 0.28	3.273 ± 2.85	0.030^a^

a *p <* 0.05. MI, Myocardial Infarction; LDL, Low Density Lipoprotein; HDL, High Density Lipoprotein; TG, Triglyceride; CK-MB, Creatine Kinase-MB; cTnI, Cardiac TroponinI.

### Cardiac troponin I is good for the later stage of CHD

Table 2 describes the comparison of cTnI levels in the blood among different groups of patients. There was no significant difference between healthy individuals and stable angina patients. The cTnI level in the blood of healthy individuals, 0.060 ± 0.00 ng/mL, was significantly lower than that of acute myocardial infarction patients, 3.273 ± 2.85 ng/mL (*p <* 0.01). The cTnI level in the blood of stable angina patients was significantly lower than that of acute myocardial infarction patients, 3.273 ± 2.85 ng/mL (*p <* 0.01). These results suggest that cTnI is more effective in diagnosing late-stage coronary heart disease (myocardial infarction) compared to early-stage detection.

**Table 2:** Comparison of the level of cTnⅠ between groups.

cTnI	Health (*n =* 48)	Angina (*n =* 59)	p
	0.060 ± 0.00	0.093 ± 0.28	0.580
	Health (*n =* 48)	MI (*n =* 62)	
	0.060 ± 0.00	3.273 ± 2.85	< 0.001*
	Angina (*n =* 59)	MI (*n =* 62)	
	0.093 ± 0.28	3.273 ± 2.85	< 0.001*

The difference between healthy individuals and MI patients is obviously, also between the stable angina patients and MI patients (*p <* 0.01).

### An increase in ANRIL is an indicator of stable angina

**Fig. 1:**
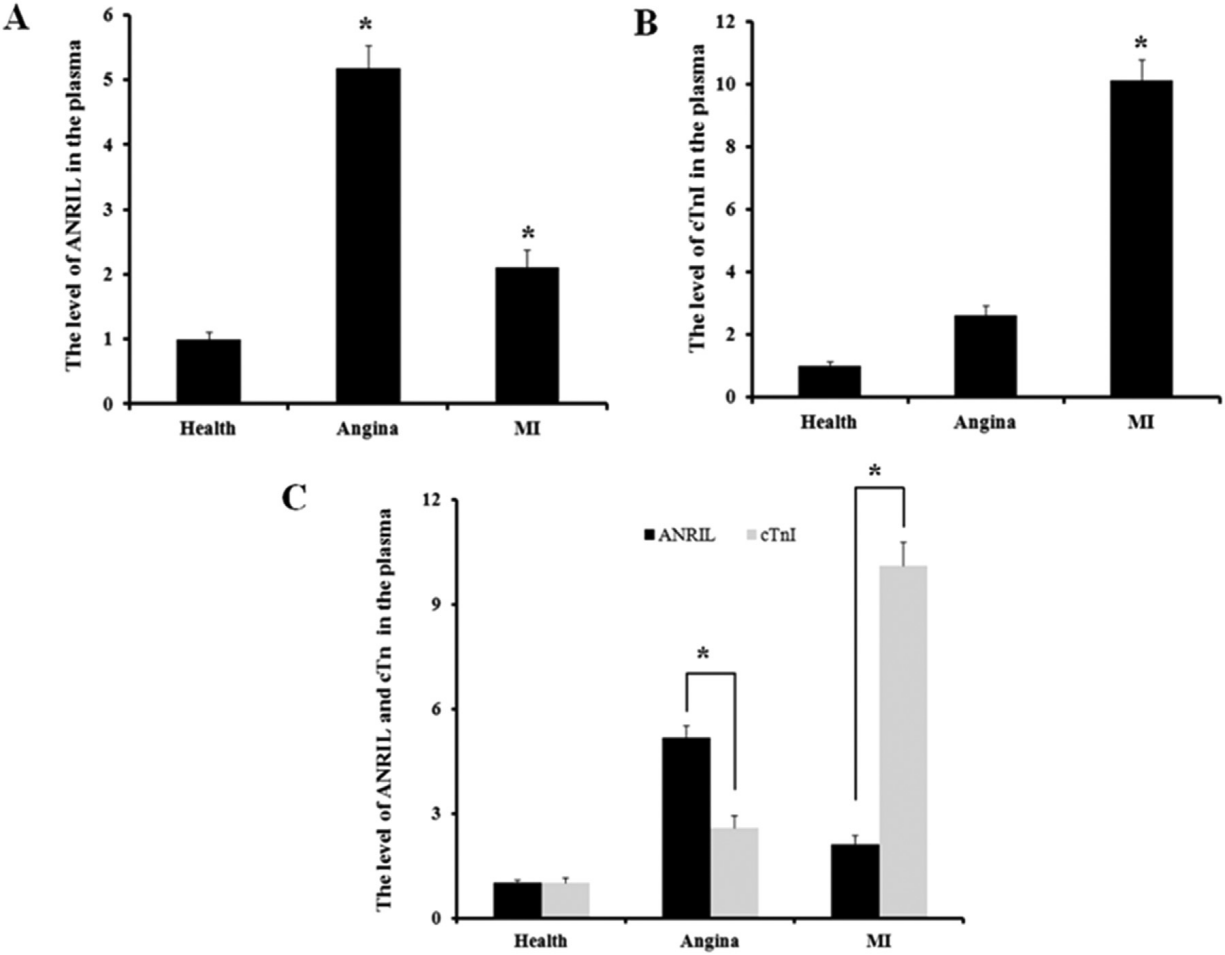
Comparison of the levels of ANRIL and cardiac troponin I in the plasma of patients with stable angina and myocardial infraction. (A) Levels of ANRIL in peripheral plasma of patients with stable angina (angina) and Myocardial Infraction (MI). (B) Levels of cardiac Troponin I (cTnI) in peripheral plasma of patients with stable angina and Myocardial Infraction (MI). (C) Comparison of cardiac Troponin I (cTnI) and ANRIL in patients with stable angina and Myocardial Infraction (MI).

ANRIL is highly expressed in the early stage of atherosclerotic plaques and may regulate the NF-κB pathway by acting as an antisense regulator to the CDKN2B-CDKN2A gene.^11^^,^^12^ However, whether the levels of ANRIL in the early stage of CHD patients’ plasma were increased was unknown. The authors analyzed the levels of ANRIL and cardiac troponin I in plasma from patients with stable angina, myocardial infarction, and healthy volunteers by real-time PCR. As shown in Fig. 1A, the levels of ANRIL in the plasma of both patients with stable angina and myocardial infarction increased significantly compared with those in the healthy individuals, but ANRIL increased much more in the patients with stable angina than with myocardial infarction. However, the levels of cardiac troponin I only significantly increased in patients with myocardial infarction (*p <* 0.05, Fig. 1B), not in patients with stable angina. Results in Fig. 1C showed that there was a significant difference (*p <* 0.05) between ANRIL and cardiac troponin I in both patients with angina patients and myocardial infarction. Notably, ANRIL increased much more than cardiac troponin I in patients with stable angina, but cardiac troponin I increased more than ANRIL in patients with myocardial infarction. These results indicate the levels of cardiac troponin I are a better index for a late stage of CHD, whereas the levels of ANRIL are a better biomarker for an early stage of CHD.

### The predictive power of ANRIL for an early stage of CHD

**Fig. 2:**
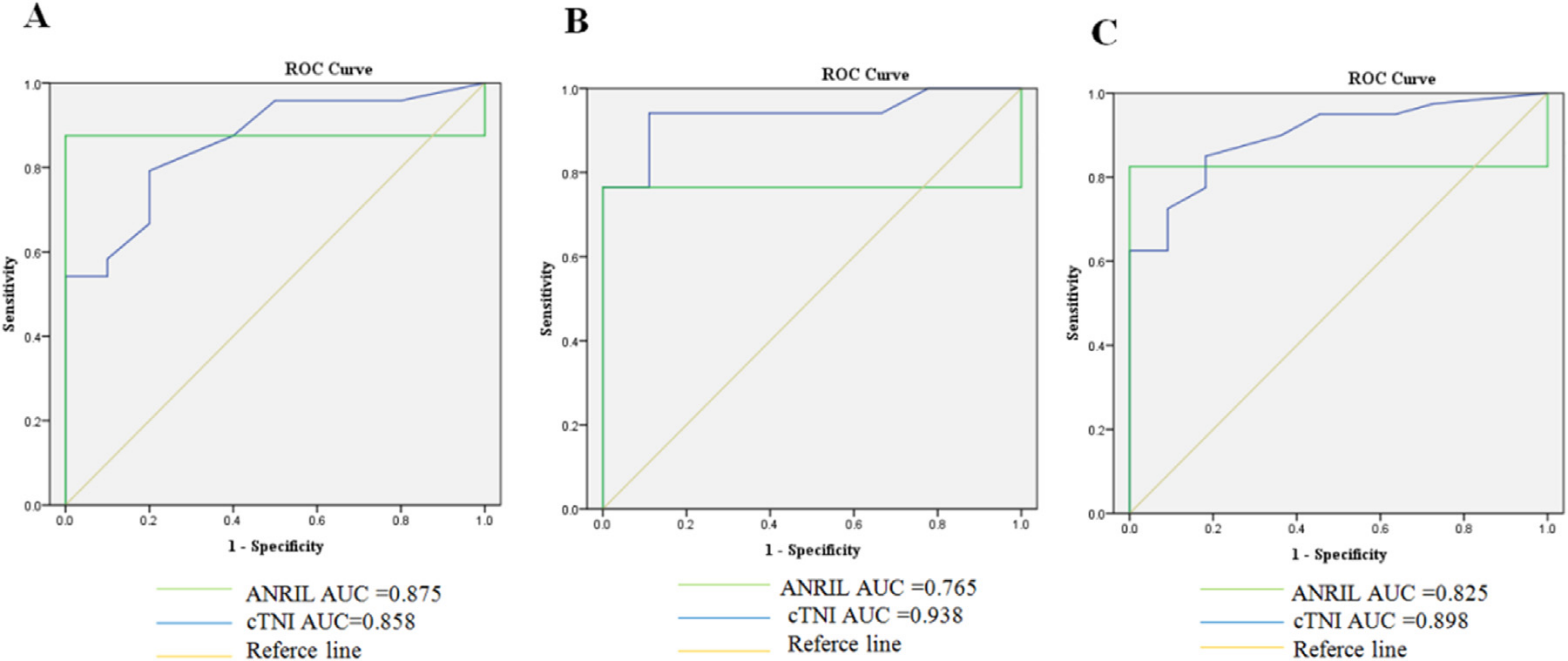
(ROC) curve plotting for ANRIL as diagnosis index. The ROC curve was analyzed to compare the accuracy of ANRIL and cardiac troponin I as diagnosis index. (A) ANRIL as the diagnosis index in patients with stable angina compared with cardiac Troponin I (cTnI), and AUC was 0.875 and 0.858 respectively. (B) ANRIL as the diagnosis index in patients with Myocardial Infraction (MI) compared with cardiac Troponin I (cTnI), and AUC was 0.765 and 0.938. respectively. (C) ANRIL as the diagnosis index in patients with stable angina and Myocardial Infraction (MI) compared with cardiac Troponin I (cTnI), and AUC was 0.825 and 0.898 respectively.

To evaluate the predictive power of ANRIL for CHD in the plasma of patients, the authors performed ROC analysis for 121 patients with CHD. As shown in Fig. 2, three ROC curves of different groups of patients were analyzed respectively. Fig. 2A shows the ROC curve for the group of patients with stable angina, the AUC of ANRIL is 0.875, and the AUC of cardiac troponin I is 0.858. These results demonstrated that ANRIL had marked sensitivity and specificity for diagnosis of stable angina. Fig. 2B describes the ROC curve for the group of patients with myocardial infarction patients, the AUC of ANRIL is 0.765, the AUC of cardiac troponin I is 0.938. The result demonstrates that ANRIL is less sensitive and specific for the diagnosis of myocardial infarction than that of cardiac troponin I. As shown in Fig. 2C, the ROC curve for the group of CHD (stable angina and myocardial infarction) patients. The AUC of ANRIL is 0.825, and the AUC of cardiac troponin I is 0.898. These results demonstrate that an increase of ANRIL in the plasma is a better indicator for an early stage of CHD than cardiac troponin I.

## Discussion

This study found a significant increase in the expression of ANRIL in the plasma of Coronary Heart Disease (CHD) patients compared to the control group. Among CHD patients, stable angina patients had a higher level of ANRIL in their plasma. ANRIL may serve as a potential circulating biomarker for the early diagnosis of CHD.

Coronary heart disease is a complex disease caused by the interaction of environmental and genetic factors. Although epigenetic changes play an important role in the development of atherosclerosis under various genetic and modifiable risk factors, data on specific gene targets of epigenetic modifications are still limited. Single molecular markers are insufficient for the diagnosis or prognosis of coronary heart disease. Given the close association between abnormal expression of ANRIL and endothelial cell injury, smooth muscle cell proliferation, migration, aging, and apoptosis, monocyte adhesion and proliferation, lipid metabolism disorders, and DNA damage, as well as its critical role in the development of atherosclerosis, ANRIL provides further insights for clinical diagnosis and prognosis. The focus of this study is to identify potential circulating biomarkers for the rapid and accurate diagnosis and monitoring of coronary heart disease through combined detection including myocardial injury markers and LncRNA.

An increasing number of studies in the literature indicate that long non-coding RNAs (lncRNAs) play important roles in many physiological and pathological processes, and several circulating lncRNAs have been applied as biomarkers for the diagnosis and prognosis of cancer.^14^, ^15^, ^16^, ^17^, ^18^, ^19^ The expression of ZFAS1, CFLAR-AS1, POU3F3, and GAS5 has been tested as biomarkers for the diagnosis and postoperative prognosis of different types of cancer.^20^^,^^21^ As one of the most extensively studied lncRNAs, ANRIL is involved in various entities, including neurological disorders, cardiovascular diseases, liver diseases, and diabetes. Studies have found that the expression of ANRIL is significantly reduced in purified Peripheral Blood T-Cells (PBTL), monocytes, and plaque tissue of individuals carrying risk alleles associated with increased risk of coronary heart disease, stroke, and aortic aneurysm.^^22^^ Ahmed W's study also demonstrated a strong association between ANRIL single nucleotide polymorphisms and myocardial infarction as well as familial hypercholesterolemia patients in a population from northern Pakistan.^23^ Holdt LM's research showed a weaker association of Chr9p21 with common cardiovascular risk factors such as lipids and hypertension.^24^ Burcu Bayoglu found that the polymorphisms on chromosome 9p21.3 may be associated with susceptibility to hypertension and coronary artery disease in the Turkish population.^25^ However, the levels of ANRIL in plasma are not yet clear. Clinical studies have shown that ANRIL accelerates the progression of Coronary Heart Disease (CHD) by regulating its Single Nucleotide Polymorphisms (SNPs). Cheng et al. found that two variants of ANRIL exons, rs10965215, and rs10738605, were associated with susceptibility to Myocardial Infarction (MI).^26^ The G allele frequency of rs10965215 increased the risk of MI, while the C allele frequency of rs10738605 increased the risk of MI. Another study indicated that ANRIL polymorphisms were associated with the risk of coronary heart disease in the Han Chinese population, suggesting an increased risk of coronary heart disease in relation to ANRIL single nucleotide polymorphism (rs10757274) in different genders.^27^ Zhuang et al. found that ANRIL rs10757274 was closely associated with the development of coronary heart disease.^28^ Samaneh et al. (100) discovered a risk allele of ANRIL single nucleotide polymorphism (rs7865618) that was associated with coronary heart disease in a cohort study.^29^ These studies suggest that ANRIL may serve as a biomarker for the diagnosis and prognosis of coronary heart disease.

This study detected the plasma circulating levels of stable angina, myocardial infarction, and healthy volunteers, and found that the circulating ANRIL levels in the plasma of coronary heart disease patients were higher than those of healthy individuals. Interestingly, the circulating ANRIL levels of patients with stable angina and myocardial infarction were significantly different: the circulating ANRIL levels in the plasma of patients with stable angina were higher than those with myocardial infarction. In addition, the authors compared the diagnostic ROC curves of circulating ANRIL levels with the traditional indicator cTnI. As a diagnostic indicator for stable angina in coronary heart disease, the AUC of circulating ANRIL was 0.875, and the AUC of cTnI was 0.858. As a diagnostic indicator for myocardial infarction in coronary heart disease, the AUC of circulating ANRIL was 0.765, and the AUC of cTnI was 0.938. As a diagnostic indicator for coronary heart disease, the AUC of circulating ANRIL was 0.825, and the AUC of cTnI was 0.898. The ROC analysis showed that ANRIL in plasma provided a more accurate diagnostic rate in the stable angina stage, suggesting that ANRIL in plasma may be more sensitive and specific for the diagnosis of early coronary heart disease.

However, there are still some limitations to this study. Firstly, a relatively small and single ethnic sample was used. Therefore, a larger sample size is needed to validate the current findings. Secondly, only the expression of ANRIL in plasma was detected, and testing for other biomarkers and/or LncRNAs may provide additional information.

In summary, the present research findings suggest a positive correlation between the expression of ANRIL in plasma and the severity of early-stage coronary heart disease.

## Conclusion

ANRIL is increased in the plasma of patients with stable angina and myocardial infarction and is a good biomarker for stable angina, an early stage of CHD.

## CRediT authorship contribution statement

**Yunjuan Jiao:** Formal analysis, Investigation, Project administration, Writing – original draft. **Fanming Meng:** Investigation. **Gaoen Ma:** Investigation, Funding acquisition, Writing – review & editing. **Hetian Lei:** Conceptualization, Funding acquisition, Writing – review & editing. **Junwen Liu:** Investigation, Conceptualization, Funding acquisition, Writing – review & editing.

## Declaration of Competing Interest

The authors declare the following financial interests/personal relationships which may be considered as potential competing interests:

Hetian Lei reports financial support was provided by National Natural Science Foundation of China.
